# Essential newborn care practices in selected public health facilities using observation of 2603 normal deliveries in Uttar Pradesh, India

**DOI:** 10.1136/bmjgh-2024-017117

**Published:** 2025-01-31

**Authors:** Shajy Isac, Bidyadhar Dehury, Ravi Prakash, Nihal Hasan, John Anthony, Banadakoppa Manjappa Ramesh, Prakash P Javalkar, Vasanthakumar Namasivayam, Marissa L Becker, James Blanchard, Ties Boerma

**Affiliations:** 1 Department of Community Health Sciences, University of Manitoba, Winnipeg, Manitoba, Canada; 2 India Health Action Trust, Lucknow, Uttar Pradesh, India; 3 Secretary, Union Territory of Ladakh, Leh, Ladakh, India

**Keywords:** Child health, Public Health, India, Hospital-based study, Health policy

## Abstract

**Introduction:**

Essential newborn care (ENBC) practices are recommended for all births to improve neonatal survival. This paper aims to understand the facility-level variations and factors associated with the essential newborn care practices by providers in higher-level public health facilities in 25 high priority districts (HPDs) of Uttar Pradesh (UP).

**Methods:**

We used observational cross-sectional quantitative data from 48 selected public health facilities (23 district hospitals (DH) and 25 community health centres (CHC)—first referral units (FRU)) implemented in 25 HPDs of UP from February 2020 to May 2021. We defined ENBC practice as both cord care and initiation of breastfeeding within 1 hour of birth were practiced in normal deliveries by the staff nurse. Descriptive analysis was done based on data from 2603 newborns attended by 318 providers. A stratified analysis was done by DH and CHC-FRU.

**Results:**

Overall, essential newborn care was practiced among 26.1% of the newborns (26.2% in DH and 35.0% in CHC-FRU). The ENBC practice varied across facilities from 3.0% to 64.1% in DH and from 0% to 91.0% in CHC-FRU. The ENBC practice was about 2.3 times higher in facilities with a high level of skill and knowledge of the providers (39.0%) compared with the facilities with a low level of skill and knowledge (16.9%). Similar patterns of association between providers’ skills and knowledge of ENBC practices were observed in DH and CHC-FRU.

**Conclusion:**

Skill and knowledge on ENBC components are significantly associated with the clinical practices of providers, with a high level of variability across facilities. This suggests a focused facility-based assessment and enhancement of the clinical competencies of the providers to improve the quality of care in public health facilities in UP.

**WHAT IS ALREADY KNOWN ON THIS TOPIC**

- Clean cord care and initiation of breastfeeding within an hour after delivery are recognised as critical interventions that significantly improve neonatal survival.
- The essential newborn care (ENBC) practices are often measured using information collected in the household surveys, which are largely affected by recall biases and measurement issues.
- Household survey data typically lacks detailed information on provider-level or facility-level care practices that can significantly influence newborn outcomes.
- In the present paper, we used facility-level observation data on delivery to better understand the provider and facility-level characteristics associated with the clinical practices of ENBC.

**WHAT THIS STUDY ADDS**

- Our study is uniquely based on the direct observation of 2603 normal deliveries in selected higher-level public health facilities in Uttar Pradesh.
- This study shows that ENBC was practiced only among one-fourth of newborns, with significant variations across facilities.
- Even within the facility, contrary to expectation, the study observed a significant variation in the practice of ENBC by the providers.
- Data linking the providers’ knowledge and skill with clinical practices are not widely available. The findings revealed a significant positive association between provider’s skills and knowledge on ENBC with ENBC practice. Moreover, facilities with higher composite scores in skills and knowledge demonstrated better adherence to ENBC practices.

**HOW THIS STUDY MIGHT AFFECT RESEARCH, PRACTICE OR POLICY**

- This study highlights the need for targeted interventions aimed at enhancing the skills and knowledge of healthcare providers both at the individual and facility- level to improve the practice of essential newborn care.
- The findings from this study can help policymakers, program planners, and key stakeholders in improving the quality of intrapartum care in low and middle-income settings.

## Introduction

 Uttar Pradesh (UP), the most populous state of India with an estimated population of 238 million by 2024,^1^ accounts for 17% of India’s population and contributes to 30% of India’s neonatal deaths and 6.7% of global neonatal deaths (author’s estimation). Recent data showed that the state observed a considerable reduction in neonatal mortality rate (NMR) from 45.1 neonatal deaths per 1000 live births in 2015–2016 to 35.7 in 2019–2020; however, the level was substantially higher than the national average of 24.9.^2^ Despite a substantial reduction in the NMR, the pace of change needs to be accelerated by more than twice the current pace to achieve the Sustainable Development Goal (SDG) of 12 neonatal deaths per 1000 live births by 2030. Care during the initial days of life is critical for a faster reduction in the NMR, as more than two-thirds of neonatal deaths occur within the first 2 days of life. WHO recommended essential newborn care practices for all births to improve neonatal survival.^3^

UP witnessed a substantial increase in institutional deliveries from 20.6% in 2005–2006 to 83.4% in 2020–2021.^2^ With increased access to institutionalised care, the demand for quality care that is safe, effective, patient-centred, timely and equitable has also increased.^4 5^ While facility births have increased over time, considerable newborn deaths occur before they are discharged from the facilities due to multiple reasons, with the most common being the low quality of care^6^ which includes low adherence to clinical guidelines in delivery care and lack of attention to essential services.^7^ WHO recognised hygienic cord care including late cord clamping performed 1–3 min after births in the correct location, cord cutting with sterile blade/scissor as well as initiation of breastfeeding within an hour as strong recommendations for all births while initiating simultaneous essential newborn care (ENBC).^3^ It was recognised that delayed cord clamping is crucial to practice in all births to improve the health outcomes of the newborn^8^ as evidence suggested that clean cord care is associated with 37% lower neonatal mortality.^9^ It is also estimated that early breastfeeding initiation could avert approximately 22% of neonatal deaths globally.^10^ The risk of neonatal deaths increases by 1.3 times when the initiation is delayed between 2 and 23 hours^11^,^13^ and it doubles when the initiation is delayed beyond 24 hours.^14^

Acknowledging the need to improve healthcare services, the Uttar Pradesh Technical Support Unit (UPTSU)—a Bill and Melinda Gates Foundation funded project implemented by the University of Manitoba and India Health Action Trust, supported Government of UP in improving the clinical practices of providers in public health facilities for better maternal and newborn outcomes through multiple innovations. A nurse mentoring intervention was introduced to improve the quality of care around birth in public health facilities through nurse mentors. The nurse mentors were a dedicated clinical staff appointed in the public health facilities to strengthen the clinical competencies—knowledge, skill and practice of staff nurses (SNs) around mother and newborn care, intrapartum and postpartum care, management of maternal and newborn complications as well as strengthening the health systems at the facility level.^15^ Similarly, the Regional Resource Training Centre (RRTC) program was initiated to improve the Comprehensive Emergency Obstetric and Newborn Care (CEmONC) services, including the management of maternal and newborn complications in district hospitals (DHs).^16^ Under the RRTC program, the faculty members of the medical colleges conducted mentoring of doctors (MBBS and specialist doctors) to strengthen the clinical competencies on CEmONC services. Along with the interventions to improve the competencies of SNs and doctors to improve the quality of health services, other health system strengthening support to the Government of UP involved strategies to improve human resources for health by bringing various policy changes related to recruitment, strengthening the procurement of logistics and medical supplies through establishing Uttar Pradesh Medical Supply Corporation Limited aimed at uplifting public health facilities to manage maternal and newborn health effectively.

The clinical practices can be driven by facility-level characteristics along with provider and individual-level characteristics. To bring a faster rate of change in neonatal mortality, programmatically, it is important to understand facility characteristics as well as provider characteristics that influence the clinical practices so that appropriate interventions can be placed. Evidence on practices of cord care, including delayed cord clamping and cutting with new blades and scissors in public health facilities in UP, is limited. Often, the essential newborn care practices are measured using the information collected from the beneficiaries in the household surveys, which are largely affected by recall biases. The household survey data also lacks information on provider-level or facility-level interventions conducted soon after the delivery. Previously, authors have also recognised measurement issues in early postnatal newborn care indicators in household surveys.^17^ In the present paper, we used facility observation data to understand the provider and facility-level characteristics associated with the clinical practices of ENBC. The study results will be useful for policymakers, program planners and key stakeholders in improving the quality of intrapartum care, with more focus on newborn care before discharge, in low and middle-income settings.

## Data and methods

### Study design and participants

This study used the facility-based cross-sectional observation data from the Rolling Facility Survey Plus (RFS+) implemented by the UPTSU from February 2020 to May 2021 in 25 high priority districts (HPDs) of UP. The HPDs were identified by the Government of India and the Government of UP based on a set of poor health indicators to ensure equity in overall health outcomes. These 25 HPDs have a total population of 69.6 million, accounting for 35% of the state population. RFS+ was uniquely designed to provide a comprehensive view of the facility to monitor the quality of care in intrapartum and immediate postpartum periods in public health facilities. The survey measured the knowledge, skills and clinical practices of SNs and auxiliary nurse midwife (ANMs) attending deliveries in DHs and community health centres (CHCs) that are first referral units (FRUs) to provide CEmONC services. Moreover, RFS+ additionally measure facility readiness in terms of nine signal functions, as well as identification and management of maternal and newborn complications.

RFS+ was conducted in 23 DHs and 25 CHC-FRUs. Two district hospitals were converted into a medical college and hence were not included in the study. One CHC-FRU was randomly selected from each of the 25 districts. A key measure of the study was the management of maternal and newborn complications as per protocol. The sample size for the RFS+ was calculated based on the probability of the management of maternal and/or newborn complications as per protocol. In the absence of any other reliable estimate, this probability was assumed to be 0.5, which would provide the maximum required sample size. The required sample of complications was calculated assuming 50% of all maternal/newborn complications are managed as per protocol and assuming a 95% CI and precision of 5%. With these assumptions, the estimated required sample of complication cases was 700. The estimated sample allocation for each facility was done based on the distribution of complications reported by each selected facility. Research investigators with clinical backgrounds were deployed at different arrival points in the facility for screening of arrivals and tracking the patient journey within the hospital until their outcomes. A framework depicting this process is provided in online supplemental figure S1. Overall, 32 553 women and newborn arrivals in the facility were screened: 8980 from the labour room, 4070 from triage, 18 485 from the outpatient department, 144 from the emergency and 874 from the special newborn care unit/newborn stabilisation unit. Overall, 10 738 met the eligibility criteria: ((1) pregnant women with ≥24 gestation period arrived in labour, (2) women arrived with postnatal complications within 42 days of delivery and (3) newborns arrived with complications within 29 days) and included in the survey. Half of these eligible cases (5368) were selected for full observation, while the remaining half (5370) underwent partial observation. However, a sequential sampling technique was used to minimise selection bias in assigning cases to full or partial observation. The full observation cases were those where a woman was observed completely from arrival to discharge—to capture the clinical practices, which included arrival patterns, initial assessment, intrapartum care including complication management and immediate postpartum care until 2 hours post-delivery (for normal deliveries) and until discharge (for complication cases). In contrast, the partial observation cases did not include the direct observation of delivery and/or complication management in the labour room. Thus, for the analysis, only cases with full observations were included. Of the 5368 cases with full observation, 5196 pregnant women met the criteria of being in labour at 24 or more weeks of gestation. Women who arrived with postpartum complications (165 cases) and newborns who arrived with complications (7 cases) were excluded from the analysis. Among the 5196 pregnant women, 3887 had normal deliveries. Women who had caesarean deliveries (444), those who did not deliver during the observation period (841) and women with incomplete clinical practice data (24) were also excluded from the analysis. Among the 3887 normal deliveries, 3790 resulted in live births and 97 resulted in stillbirths. In the case of twins/triplets, observation was done for the firstborn newborn. Among 3790 live births, 503 newborns were identified with any complications including birth asphyxia, preterm, birth weight <1800 g, sepsis and birth anomaly (503 cases). Since ENBC was not observed for these cases, they were excluded from the analysis. During the analysis, information from multiple schedules was merged into the delivery observation data and thus, 684 observations with missing information were dropped from the analysis. Therefore, the overall analysis was based on 2603 newborns (1860 in DH and 743 in CHC-FRU) conducted by 318 providers (192 in DH and 126 in CHC-FRU) from selected 48 public health facilities.

### Survey questionnaire

The survey canvased nine distinct schedules, each aimed at capturing different domains crucial to maternal and newborn care. These domains included facility readiness, arrival patterns, staff clinical skills and knowledge, practices during normal deliveries, management of maternal and newborn complications, pre-referral procedures and immediate health outcomes. The study assessed clinical practices through direct observation of deliveries, assessed staff skills through demonstrations at skill stations and assessed their knowledge through interviews. Handheld mobile phones/tablets equipped with Open Data Kit-based android applications were used for data collection during the observations/interviews. These applications featured automatic skipping patterns, outlier detection and reminders to enhance data accuracy and reduce the need for manual checks. Completed forms were submitted directly from the field to a web-based server, enabling real-time data management and minimising the risk of data loss.

### Outcome variable

A composite outcome indicator of ENBC was computed based on two critical components of recommended essential newborn care–cord care and early initiation of breastfeeding (EIBF). The ENBC practice is defined as both cord care, and EIBF practiced in normal deliveries by the providers. Cord care is defined as the cord being clamped after 1–3 min of birth (late clamp), clamped in the correct location and cut with a sterile blade/scissor. The EIBF is defined as the breastfeeding was initiated within 1 hour after birth. The selection of these two ENBC components for analysis was guided by preliminary findings, which revealed that certain ENBC components, like weighing, immediate drying and wrapping the newborn, had high coverage levels (over 90%). In contrast, cord care (clamping and cutting) and early initiation of breastfeeding showed relatively lower coverage levels. Based on these insights, ENBC components with lower coverage were chosen for further analysis, focusing on areas with potential for improvement to inform targeted interventions where they may have the greatest impact.

### Explanatory variables

A set of individual, provider and facility-level covariates were included in the analyses. Background characteristics of women (age, education, gravida, religion, caste, residence) and time of birth were included in the analysis. Background characteristics of women were obtained in the study by asking relevant questions to the respondents. The actual time of birth was recorded by the researchers during the observation, and for analysis purposes, we coded them as ‘8 am – 2 pm’, ‘2 pm – 8 pm’ and ‘8 pm – 8 am’. At the provider level, their experience, skill and knowledge of ENBC were considered. Provider’s work experience was grouped as <5 years, 5–9 years and 10+ years. Provider’s skills in ENBC were assessed through the demonstration of ENBC components at the skill station during the survey, and a set of questions was administered among the providers to assess their knowledge in the ENBC domain. Three indicators were used in computing the skill: (1) clamped and cut the umbilical cord in 1–3 min, (2) encouraged the initiation of breastfeeding with the support of other staff and birth companion and (3) encouraged the initiation of breastfeeding as early as possible within 1 hour of birth. Two indicators were used to compute knowledge: (1) know ‘clamp and cut the cord within 1–3 min’ as a component of ENBC, and (2) know ‘initiate breastfeeding with skin-to-skin contact’ as a component of ENBC. A combined ‘skill and knowledge’ score was computed based on these five indicators. The providers were grouped into three categories based on the obtained score out of a total of 5: low (0–2), medium (3) and high (4–5). At the facility level, monthly per-capita delivery load by staff in the facility, level of facility (DH or CHC-FRU), availability of equipment (‘cord clamp or sterile thread’ and ‘scissors’) and collective skill and knowledge score in ENBC were considered. Facilities were grouped into three categories based on the facility level skill and knowledge score; low (<70%), medium (70–79%) and high (≥80%).

### Statistical analysis

We developed an analytical framework to assess the association of individual, provider and facility-level characteristics on the clinical practices of ENBC (online supplemental figure S2). Univariate analysis was conducted to describe the profile of the deliveries observed, providers’ characteristics and facility characteristics. Bivariate analysis was conducted to understand the differentials in the practice of ENBC by individual, provider and facility-level characteristics. Facility-level heterogeneity in the practice of ENBC was presented across 18 DHs and 19 CHC-FRUs. 5 DHs and 6 CHC-FRUs with observations of less than 20 were dropped in facility-level analysis. A stratified analysis was also done by type of facility (DH and CHC-FRU) to understand the differentials in ENBC practices by level of care. The analysis was done in Stata V.16.

## Results

### Profile

Of 2603 normal deliveries observed, 28.0% of deliveries were conducted between 08:00 and 14:00, 23.6% between 02:00 and 20:00 and almost half of the deliveries (48.4%) were conducted during night shifts between 20:00 and 08:00 (online supplemental table S1). Of the total 318 providers included in the study, the average work experience was 9.3 years (9.0 years in DH and 9.8 years in CHC-FRU), with about one-third of providers (34.3%) having 10 years or more of work experience. While 62.9% providers had high levels of skills and knowledge, 16.7% of providers had low and 20.4% of providers had a moderate level of skill and knowledge on ENBC. Among 48 facilities, the required equipment for ENBC (‘Cord clamp or sterilized thread’ and ‘scissors’) was available in 81.2% of the facilities. Per-capita monthly delivery load per staff was 17.6 in CHC-FRU and 11.3 in DH. Overall, 60.4% of facilities had an average monthly delivery of less than 15 deliveries per staff, 12.5% of facilities had 30+ and 27.1% had a 15–30 average monthly delivery load per staff. Overall skill and knowledge score of ENBC at the facility level was low in one-fifth of the facilities, moderate in 41.7% of facilities and high in 37.5% of facilities. A higher proportion of CHC-FRUs (44.0%) had high skill and knowledge scores compared with DHs (30.4%).

### Differentials in ENBC practice

Of the 2603 normal deliveries observed, ENBC, as defined as both cord care and EIBF was practiced in 26.1% (95% CI: 24.4%, 27.8%) of newborns (table 1). Cord care was practiced in 45.7% (95% CI: 43.8%, 47.6%) of newborns (where all three components of cord care were practiced), and EIBF was practiced in 46.7% (95% CI: 44.8%, 48.6%). ‘Delayed cord clamping’ and ‘cord cutting using a new blade/ sterile scissor’ were practiced among two-thirds of newborns, while ‘cord clamping in correct location’ was practiced among 90.5% (95% CI: 89.3%, 91.6%) of newborns. The results showed differences in the practice of ENBC by facility type (35.0% (95% CI: 31.6%, 38.4%) in CHC-FRU and 22.6% (95% CI: 20.7%, 24.5%) in DH). The practice of cord care and EIBF was higher in CHC-FRU (55.6% (95% CI: 52.0%, 59.2%) and 54.5% (95% CI: 50.9%, 58.1%)) as compared with the DH (41.7% (95% CI: 39.5%, 44.0%) and 43.5% (95% CI: 41.3%, 45.8%)), respectively. Among the cord care components, ‘delayed cord clamping’ was observed to be higher in CHC-FRU (82.0% (95% CI: 79.2%, 84.7%)) as compared with that in DH (61.3% (95% CI: 59.1%, 63.5%)). Differentials in ENBC practices by individual level characteristics are available in online supplemental table S2.

**Table 1:** Percentage of essential newborn care practices stratified by type of facility

Facility type	Overall, % (95% CI)	DH, % (95% CI)	CHC-FRU, % (95% CI)
Clamped cord in correct location	90.5 (89.3, 91.6)	88.7 (87.3, 90.1)	94.9 (93.3, 96.5)
Delayed cord clamping	67.2 (65.4, 69.0)	61.3 (59.1, 63.5)	82.0 (79.2, 84.7)
Cord cutting using new blade/sterile scissors	68.0 (66.2, 69.8)	69.5 (67.4, 71.6)	64.3 (60.9, 67.8)
Cord care (all three components)	45.7 (43.8, 47.6)	41.7 (39.5, 44.0)	55.6 (52.0, 59.2)
EIBF	46.7 (44.8, 48.6)	43.5 (41.3, 45.8)	54.5 (50.9, 58.1)
ENBC (cord care and EIBF)	26.1 (24.4, 27.8)	22.6 (20.7, 24.5)	35.0 (31.6, 38.4)
Number (# of live births)	2603	1860	743

CHCcommunity health centresDHdistrict hospitalEIBFearly initiation of breastfeedingENBCessential newborn care FRUfirst referral unit

Differentials in the practice of ENBC are presented by individual-level, provider-level and facility-level characteristics stratified by facility type (table 2). The practice of ENBC was higher among the newborns delivered during the daytime (28.3% between 08:00 and 14:00 and 27.5% between 14:00 and 20:00) compared with the deliveries during the night shift (24.2% between 20:00 and 08:00) with significant differences in DH. The practice of ENBC varied significantly according to the level of skill and knowledge of the providers. For example, the practice of ENBC was higher among the providers with high skill and knowledge (27.9%) or the providers with medium skill and knowledge (25.9%) compared with the providers with low level of skill and knowledge (17.8%). This holds true for both DH and CHC-FRU, with providers having higher levels of skills and knowledge (23.7% in DH and 38.2% in CHC-FRU) compared with those providers with lower levels of skills and knowledge (15.2% in DH and 23.5% in CHC-FRU). ENBC practice was 33.2% in the facilities with a lower monthly per-capita delivery load (<15 deliveries per provider in a month) compared with 12.1% in the facilities with 30 or more deliveries per provider. Similar patterns were observed in DH (27.0% vs 6.9%) and CHC-FRU (48.5% vs 26.4%). The practice of ENBC also varied by facility level skill and knowledge score. Newborns delivered in facilities with high composite scores of skill and knowledge had almost twice (39.0%) ENBC practices as compared with the newborns delivered in facilities with low or medium composite scores (16.9% and 21.4%, respectively). Similar patterns were observed for DH (16.0% in facilities with low skill and knowledge compared with 39.0% in facilities with high skill and knowledge) and CHC-FRU (19.7% vs 39.1%, respectively).

**Table 2:** Percentage of essential newborn care practices by individual-level, provider-level and facility-level characteristics stratified by facility type

Characteristics	Overall		DH		CHC-FRU	
	% (95% CI)	n	% (95% CI)	n	% (95% CI)	n
Overall	26.1 (24.4, 27.8)	2603	22.6 (20.7, 24.5)	1860	35.0 (31.6, 38.4)	743
Individual level						
Timing of delivery						
08:00 – 14:00	28.3 (25.0, 31.6)	728	26.5(22.7, 30.3)	513	32.6 (26.3, 38.8)	215
14:00 – 20:00	27.5 (24.0, 31.0)	615	22.1 (18.3, 26.0)	443	41.3 (33.9, 48.6)	172
20:00 – 08:00	24.2 (21.8, 26.6)	1260	20.6 (17.9, 23.2)	904	33.4 (28.5, 38.3)	356
Provider level						
Work experience						
<5 years	23.3 (20.0, 26.6)	618	20.4 (16.7, 24.2)	450	31.0 (24.0, 37.9)	168
5–9 years	28.2 (25.5, 30.9)	1089	25.0 (22.1, 27.9)	848	39.4 (33.2, 45.6)	241
10 years+	25.6 (22.7, 28.4)	896	20.6 (17.3, 24.0)	562	33.8 (28.8, 38.9)	334
Skill and knowledge						
Low	17.8 (13.9, 21.7)	371	15.2 (10.8, 19.6)	256	23.5 (15.7, 31.2)	115
Medium	25.9 (22.0, 29.8)	475	23.9 (19.5, 28.3)	360	32.2 (23.6, 40.7)	115
High	27.9 (25.8, 30.0)	1757	23.7 (21.4, 26.1)	1244	38.2 (34.0, 42.4)	513
Facility level						
Monthly per-capita delivery load						
<15	33.2 (30.1, 36.2)	911	27 (23.6, 30.4)	649	48.5 (42.4, 54.5)	262
15–30	29.9 (27.0, 32.8)	974	30.5 (27, 33.9)	686	28.5 (23.3, 33.7)	288
30+	12.1 (9.7, 14.5)	718	6.9 (4.7, 9.0)	525	26.4 (20.2, 32.6)	193
Availability of equipment*						
No	66.7 (61.6, 71.8)	327	77.1 (70.2, 84.1)	140	58.8 (51.8, 65.9)	187
Yes	68.2 (66.3, 70.1)	2276	68.9 (66.7, 71.1)	1720	66.2 (62.3, 70.1)	556
Total	68.0 (66.2, 69.8)	2603	69.5 (67.4, 71.6)	1860	64.3 (60.9, 67.8)	743
Skill and knowledge						
Low	16.9 (13.8, 20.1)	537	16.0 (12.4, 19.6)	400	19.7 (13.0, 26.4)	137
Medium	21.4 (19.1, 23.7)	1231	18.2 (15.8, 20.5)	1024	37.2 (30.6, 43.8)	207
High	39.0 (35.7, 42.4)	835	39.0 (34.4, 43.6)	436	39.1 (34.3, 43.9)	399

* Availability of equipment is defined as the availability of ‘cord clamp or sterile thread, and scissors’. Outcome considered for available equipment: Cord cutting using new a blade/sterile scissor.

CHCcommunity health centreDHdistrict hospitalFRUfirst referral units

**Figure 1:**
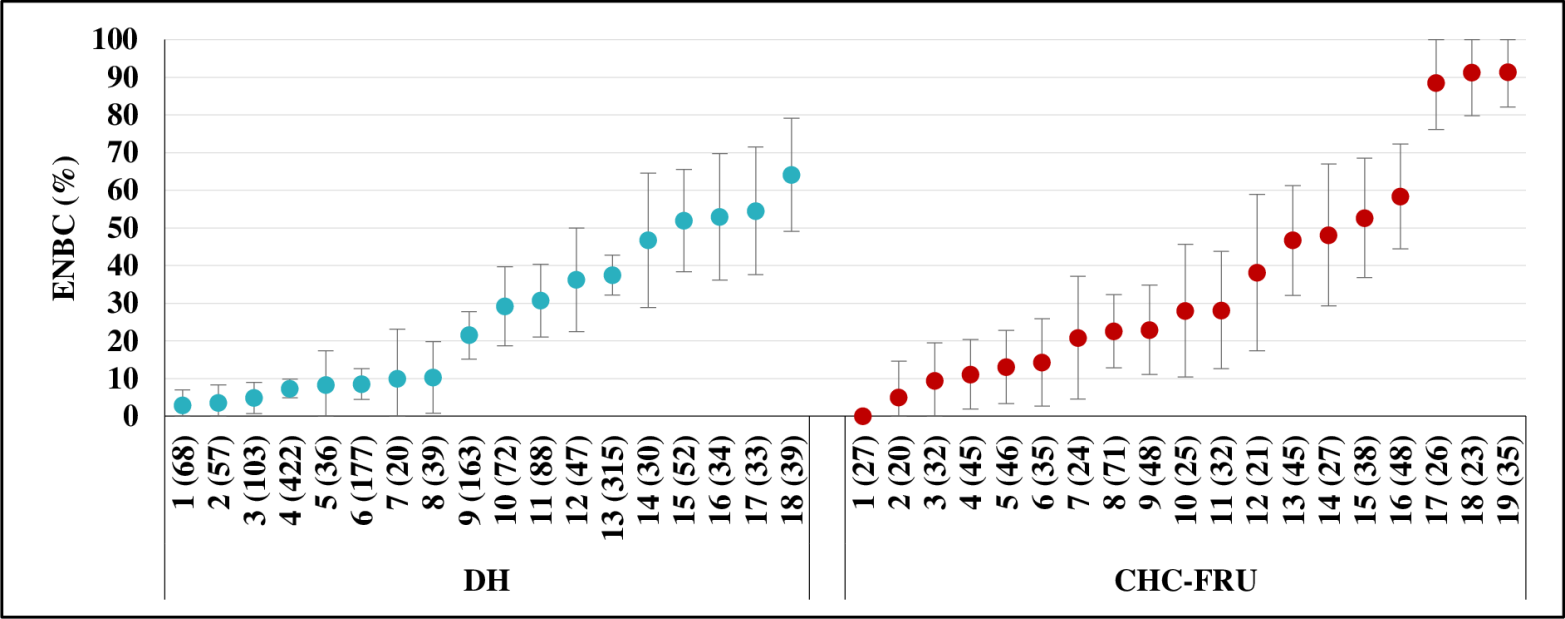
Facility level heterogeneity in the practice of essential newborn care (95% CI) across facilities by type. Note: the x-axis displays facility serial number, sorted by ENBC %, with the number of newborns shown in the brackets. CHC, community health centre; DH, district hospital; ENBC, essential newborn care; FRU, first referral units.

**Figure 2:**
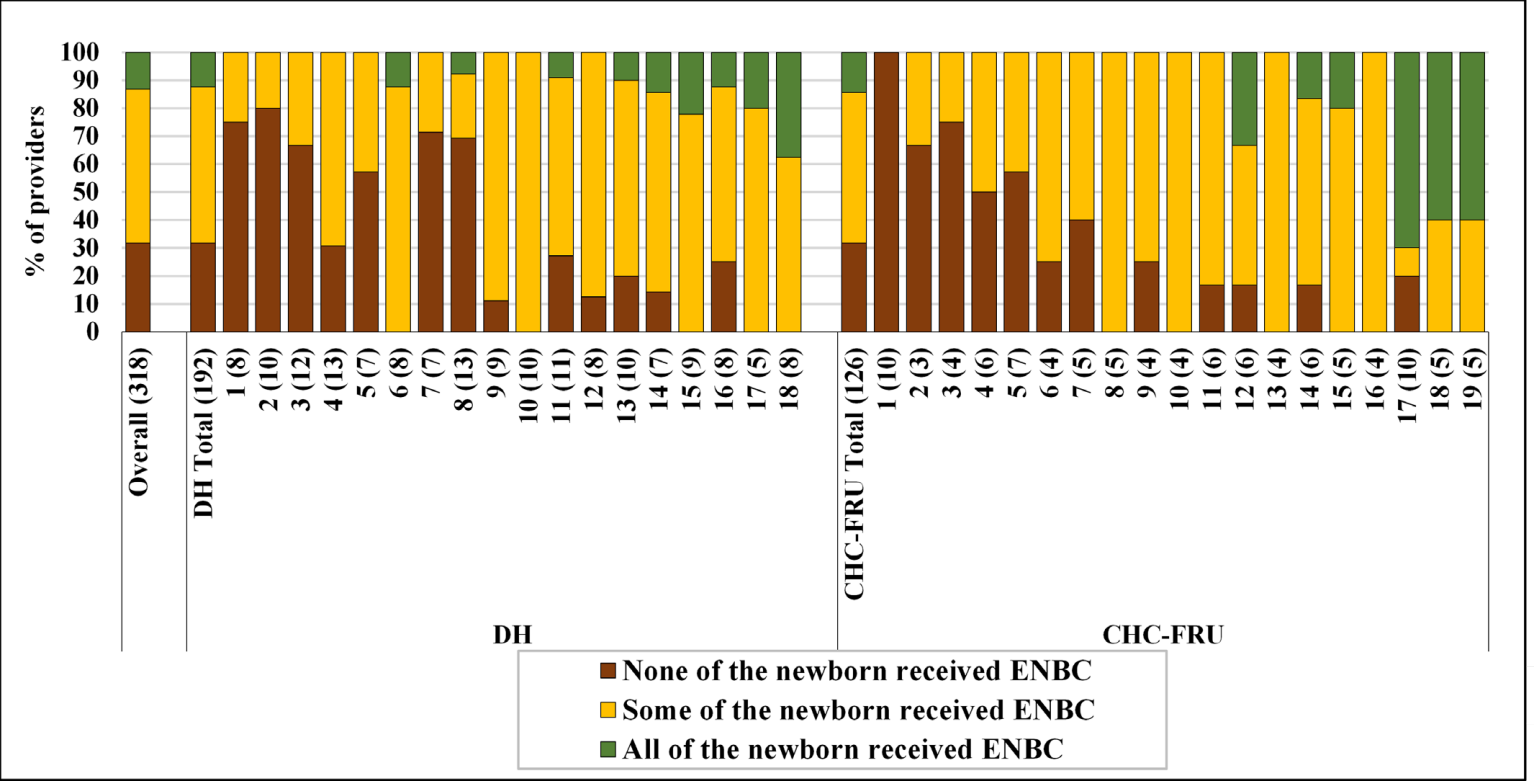
Distribution of providers according to the practice of essential newborn care across facilities. Note: the x-axis displays facility serial number, sorted by ENBC %, with the number of providers shown in the brackets. CHC, community health centre; DH, district hospital; ENBC, essential newborn care; FRU, first referral units.

**Figure 3:**
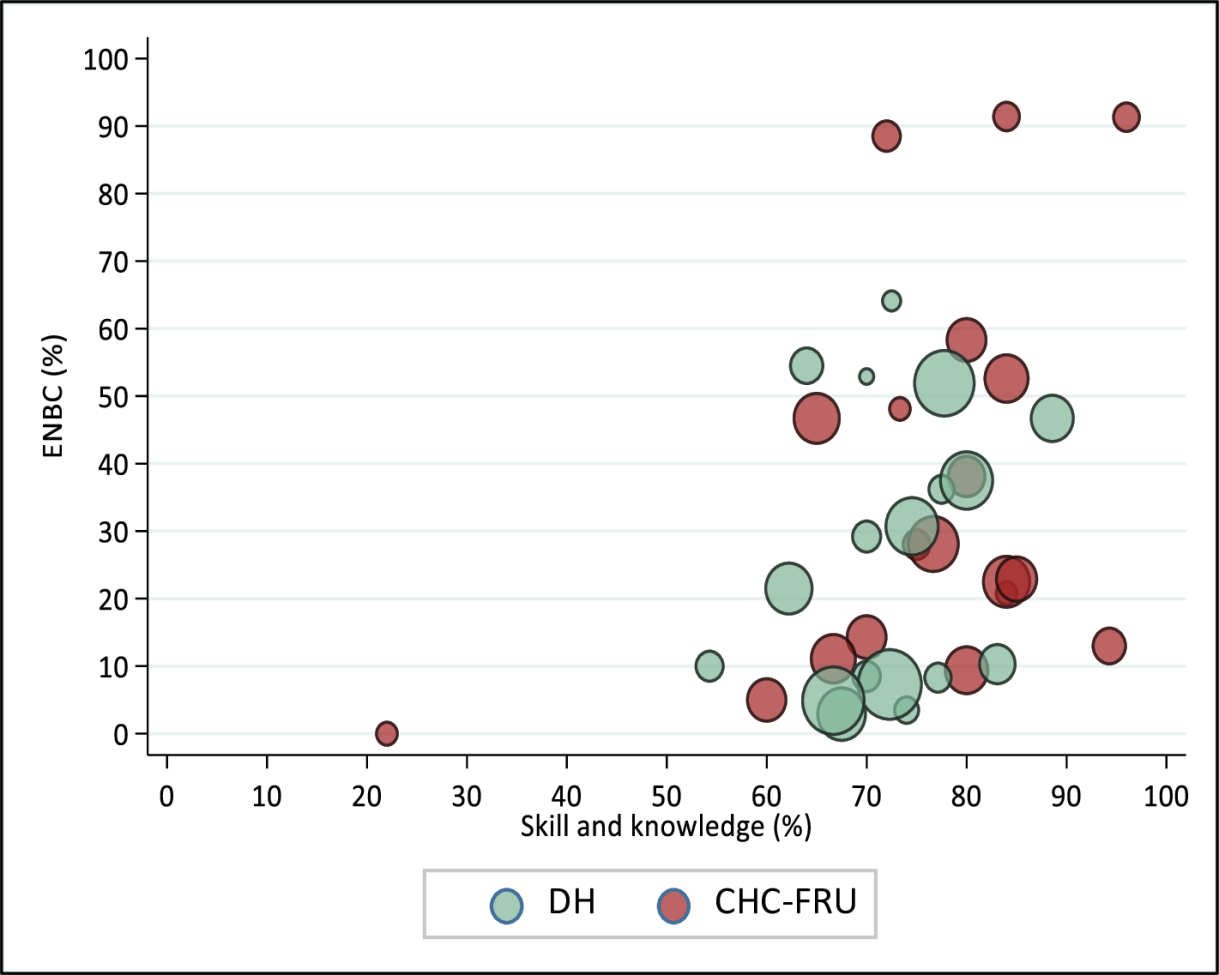
Facility-level scatter plot of skill and knowledge and practice of essential newborn care. CHC, community health centre; DH, district hospital; ENBC, essential newborn care; FRU, first referral units.

The study also found large facility-level heterogeneity in ENBC practices, ranging from 3.0% to 64.1% in 18 DHs and from 0% to 91.0% in 19 CHC-FRUs (figure 1). In DH, there were eight facilities where <20% of newborns received ENBCs, six facilities where 20–50% of newborns and four facilities where more than half of the newborns received ENBC. Similarly, in CHC-FRU, there were six facilities where <20% newborns, eight facilities where 20–50% newborns and five facilities where more than half of the newborns received ENBC. Further, we also explored ENBC practices at the provider level according to their ENBC coverage grouped as ‘none’ (did not practice during any of the delivery observed), ‘some’ (practiced in some of the deliveries observed) and ‘all’ (practiced in all of the delivery observed). Out of 318 providers, 31.8% of providers practiced ENBC in ‘none’ of the newborns, 55.0% of providers in ‘some’ of the newborns and 13.2% of providers practiced ENBC in ‘all’ of the newborns they attended (figure 2). The distribution of providers according to the practice of ENBC did not vary much between DH and CHC-FRU and followed the overall pattern. Across facilities, ‘none’ was 0% in five DHs and seven CHC-FRUs, <50% in seven DHs and seven CHC-FRUs and ≥50% in six DHs and five CHC-FRUs. The association of skill and knowledge score and ENBC practice at the facility level was examined through the scatter plot (figure 3). The size of the circle represents the monthly delivery load of the facility. The scatter plot shows a positive correlation between skill and knowledge and ENBC practice with a correlation coefficient of 0.37.

## Discussion

Using data from the direct observations of deliveries and newborn care, this paper provides a unique opportunity to assess the coverage and quality of ENBC practices in the public health settings of UP. Overall, ENBC was practiced in only one-fourth (26.1%) of the newborns by the providers in selected public health facilities in UP. Also, a wide variation in the practice of ENBC across selected public health facilities was observed. ENBC practice was found to be higher among providers with higher levels of skills and knowledge on ENBC compared with the providers with lower levels of skills and knowledge. Moreover, we found a positive association between facility-level composite scores in skills and knowledge and levels of ENBC practice.

In the recent past, the state of UP experienced an increased shifting of births from home delivery to facility-based deliveries, where more than 84% of births were conducted in facilities, with 57% in a public health facility. With increasing access to public health facilities for delivery care, it becomes important to strengthen the public health facilities in providing quality care. Our findings bring important programmatic insights that will help in designing programmes to improve the quality of care services in public health facilities in the state. The skill and knowledge in ENBC components at the provider level as well as at the facility were critical in improving the clinical practices. Using a community-level study, Namasivayam *et al* found that the likelihood of receiving breastfeeding within an hour increased by almost twofold when the mother received immediate postnatal support on breastfeeding.^18^ Our study’s results showed that ENBC practice was 1.6 times higher among providers with a high level of skill and knowledge compared with providers with a low level of skill and knowledge. We also found that ENBC practice was 2.3 times higher in facilities with a high average score of skill and knowledge compared with facilities with a low average score of skill and knowledge.

Our findings also showed differences in ENBC practice according to delivery time. The ENBC practice was higher among newborns delivered in the daytime compared with the newborns delivered during the night-time, particularly in the DHs. This indicates the challenges in receiving quality care at night. It could be because in most of the cases, women who delivered at night were approached in the morning by the provider.^19^ In addition, work experience and per-capita delivery load in CHC-FRU were significantly associated with the clinical practices of ENBC.

The study also brings an important insight into the differentials in ENBC practices within the facility. Findings revealed that 31.8% of providers practiced ENBC in none of the cases they attended, whereas 55.0% of providers practiced ENBC in some cases but not all of the cases that they attended and 13.2% of providers practiced in all of the cases that they attended appeared to be important to ensure equitable quality of care. Enhancing the skills and knowledge of providers who practiced ENBC in none of the cases is critical as they had significantly low skill and knowledge scores (66.3%) compared with those who practiced in some (76.1%) and all (80.0%) cases. In addition to the skills and knowledge level, the varying level of practice among providers who practiced ENBC in some but not all cases could be attributed to their attitudes and the enabling environment of facilities, which were identified in other studies as some of the key determinants of improved clinical practices.^20^

Studies from UP and other states of India have identified several challenges at the provider and facility level in providing quality intrapartum and immediate postpartum care services which include insufficient physical infrastructure, irregular water and electricity supply, shortages of medicines and supplies, non-availability of laboratory and diagnostic services at night, difficulties in managing complications at night due to a shortage of specialists, inadequate facilities for managing newborn complications, difficulty in maintaining privacy and inadequate facilities for managing newborn complications.^21^,^23^ A study conducted in 2017 across 24 districts in 60 pairs of public health facilities in UP reported that women and their families often faced non-availability and negligence of staff in public health facilities.^24^ Person-centred care is a key component of overall newborn health quality, and therefore, the provider’s knowledge and skill play an important role in ensuring the quality of care at the facility and influencing adherence of beneficiaries to medical advice post partum as well as their care-seeking after discharge. However, most of the previous studies were not designed to link the providers’ knowledge and skills with the actual practices in the same facility. Specific interventions at the facility were largely missing as interventions were not prioritised according to the identified gaps. The present study is an important contribution to examining the role of these factors in a holistic way and identifying the specific intervention areas requiring specific attention.

This study has a few limitations to note. The study findings are based on the data from selected public health facilities (25 out of 75 districts) of UP. The findings are based on the cross-sectional study, hence, precluding any causal interpretation of the intervention and practice of ENBC. The analysis only included observation of normal delivery and excluded complication cases, considering that the ENBC practices among newborns delivered via caesarean section may vary from those of normal deliveries. The study was implemented in higher-level facilities (DHs and CHC-FRUs) and excluded the lower-level facilities such as CHC non-FRUs and primary health centres, which cater to one-third of the total institutional deliveries. The study did not include some of the important factors, such as the healthcare provider’s personal attitude and facility-level enabling factors, that could influence the practice of ENBC. However, the results of this study have relevant policy implications. The study findings are generalisable for the entire state of UP considering the nature of the intervention. Further research is required to understand the provider’s attitude towards their work, motivational factors and facility-level enabling factors in improving appropriate ENBC practices in the public health facilities in UP.

## Conclusion

Using data from selected public health facilities in 25 HPDs of UP, this study underscores the critical role of healthcare providers, particularly SNs/ANMs, in ensuring the effective implementation of ENBC practices in public health facilities in UP. The study demonstrated facility, provider and individual level differentials in the practice of ENBC highlighting the need for targeted interventions to enhance the skills and knowledge of healthcare providers. Given that most of the normal deliveries in health facilities are conducted by SNs/ANMs, continued efforts to uplift the skills of these cadres of health professionals become imperative. Strengthening their skills not only improves the quality of care provided during childbirth but also enhances the likelihood of adherence to recommended ENBC protocols. By focusing on these cadres of health professionals, healthcare systems can effectively address gaps in ENBC practices and contribute to better maternal and newborn health outcomes in UP.

As a next step, the ongoing health system interventions should remain an area of focus in the state. While the training of existing and newly appointed SNs/ANMs is ongoing, it is equally important to periodically monitor their competencies and identify the specific areas that need re-orientation/capacity building. Creating a robust competency dictionary for healthcare providers and periodic evaluation of the knowledge and skills of the providers involved in conducting deliveries would be an important step in this regard. This would enable the assessment of all the SNs/ANMs using a standard tool and protocol. Digital applications can be used for the same, considering the huge pool of human resources (about 68 000 nursing staff and ANMs) in the state. More importantly, it would be crucial to standardise the mentoring tools/materials so that all the staff are trained using a standard, easy-to-understand approach. Routine identification of gaps and targeted interventions are needed to improve maternal and newborn health outcomes, thereby accelerating the rate of change of decline in maternal and newborn mortality and achieving the SDGs.

## supplementary material

10.1136/bmjgh-2024-017117 online supplemental file 1

## Data Availability

Data are available upon reasonable request.
