# Supplementary material for: Essential newborn care practices in selected public health facilities using observation of 2603 normal deliveries in Uttar Pradesh, India
Source: BMJ Glob Health. 2025 Jan 31;10(1):e017117. doi: 10.1136/bmjgh-2024-017117 (PMC11792291; doi:10.1136/bmjgh-2024-017117)
Supplement: online supplemental file 1 [file bmjgh-10-1-s001.pdf]

Supplements

Figure S1: Framework of sample selection

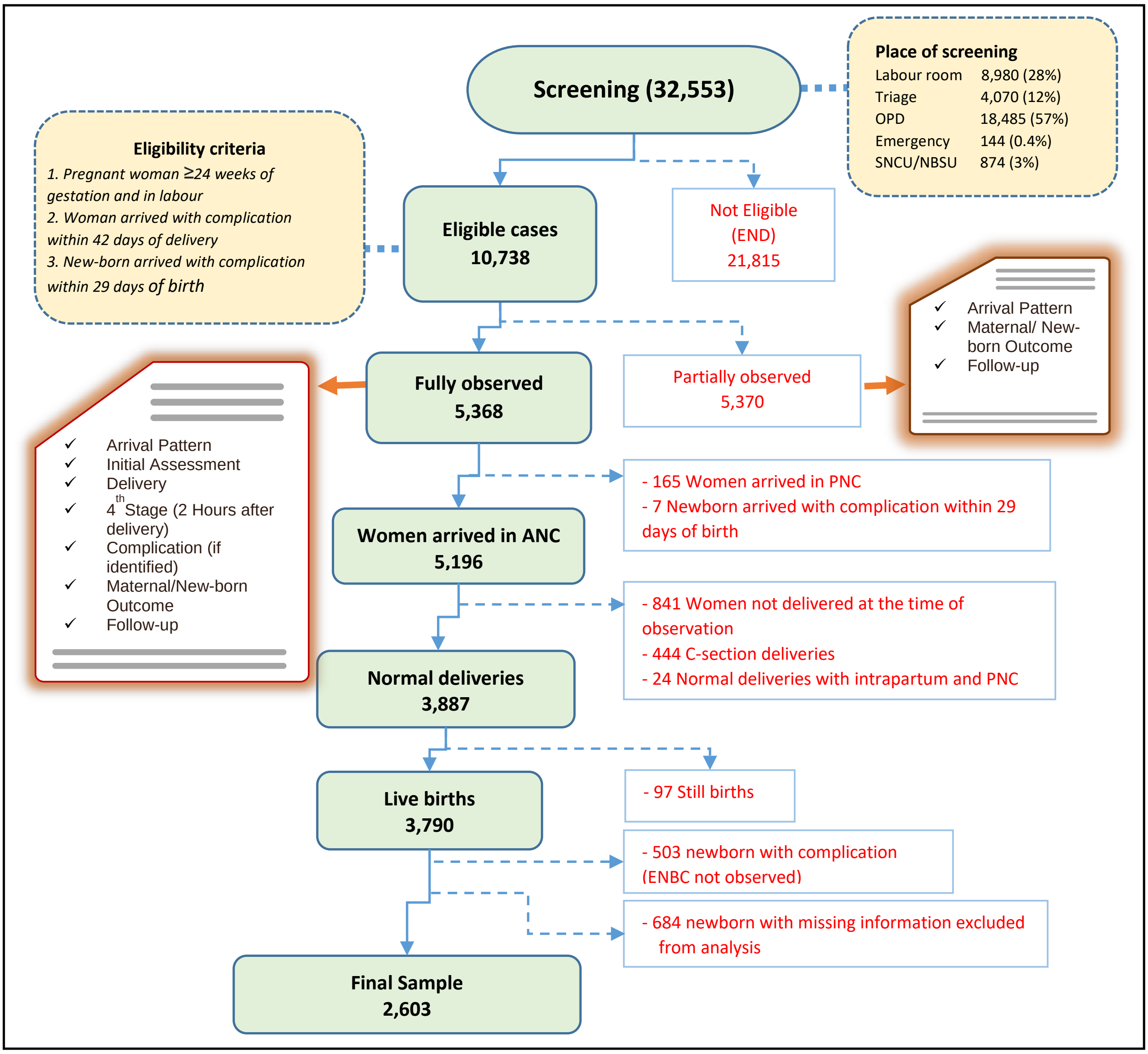

Figure S2: Analytical framework for the analysis of ENBC

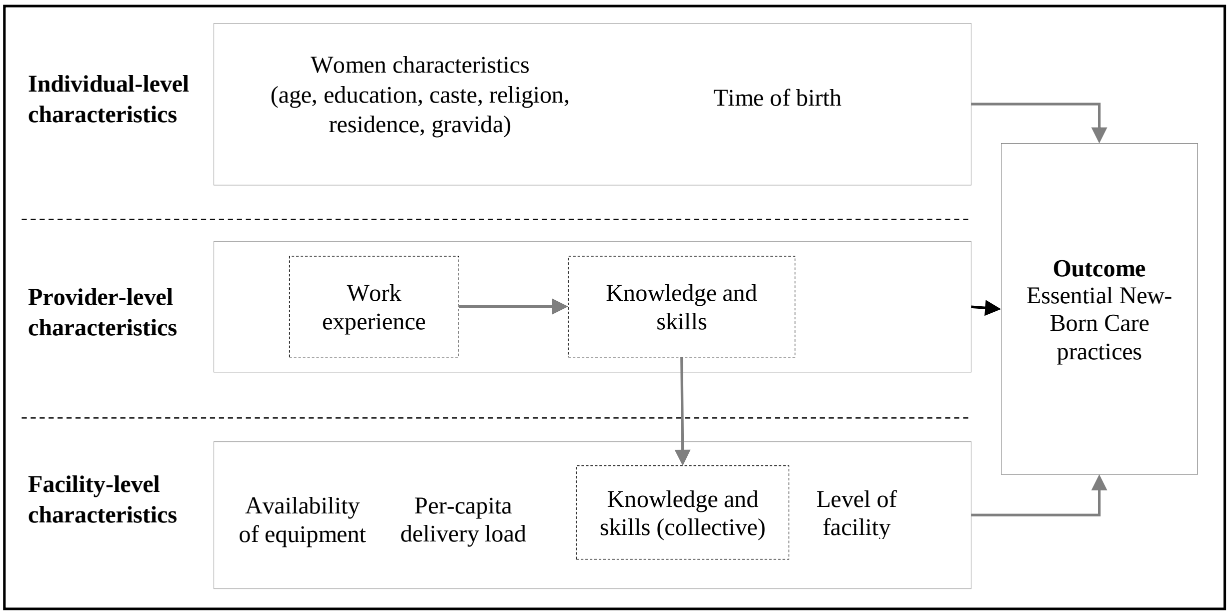

Table S1: Profile of the sample stratified by type of facility

Characteristics	Overall (%)	DH (%)	CHC-FRU (%)
**Individual level**			
Timing of delivery			
8 am-2 pm	28	27.6	28.9
2 pm-8 pm	23.6	23.8	23.2
8 pm-8 am	48.4	48.6	47.9
Total	100	100	100
Age			
<24 years	39	39.8	36.7
25-29 years	44	43.3	45.8
30+ years	16.9	16.8	17.1
Missing	0.2	0.1	0.4
Total	100	100	100
Religion			
Hindu	80.9	81.7	79
Non-Hindu	18.9	18.2	20.6
Missing	0.2	0.1	0.4
Total	100	100	100
Caste			
SC/ST	26.6	26.1	27.7
OBC	48.9	48	51.1
Other	22.9	24.4	19.3
Missing/DK	1.6	1.5	1.9
Total	100	100	100
Education			
<5 years	37.7	35.3	43.7
5-10 years	25.8	26.4	24.2
10+ years	36.3	38.2	31.5
DK/Missing	0.2	0.1	0.5
Total	100	100	100
Gravida			
1	29.1	30.3	26.1
2	30	30.9	27.7
3	23.9	23.2	25.8
4+	16.8	15.5	19.8
DK/Missing	0.2	0.1	0.5
Total	100	100	100
N (# of live births)	*2603*	*1860*	*743*
**Provider level**			
Work experience			
<5y	27	27.6	26.2
5-9y	38.7	41.2	34.9
10y+	34.3	31.3	38.9
Total	100	100	100
Mean	*9.3*	*9*	*9.8*
Skill and knowledge			
Low	16.7	14.6	19.8
Medium	20.4	22.9	16.7
High	62.9	62.5	63.5
Total	100	100	100
Mean	*73.5*	*73.9*	*73*
N (# of providers)	*318*	*192*	*126*
**Facility level**			
Monthly per-capita delivery load			
<15	60.4	69.6	52
15-30	27.1	21.7	32
30+	12.5	8.7	16
Total	100	100	100
Mean	14.6	11.3	17.6
Availability of equipment[1]			

No	18.8	17.4	20
Yes	81.2	82.6	80
Total	100	100	100
Skill and knowledge			
Low	20.8	26.1	16
Medium	41.7	43.5	40
High	37.5	30.4	44
Total	100	100	100
Mean	*73.5*	*73.9*	*73*
N (# of Facilities)	*48*	*23*	*25*

Note: [1]Availability of equipment is defined as availability of "cord clamp or sterile thread, and scissors"

Table S2: Percentage of essential newborn care by individual characteristics

Characteristics	Coverage (%)					
	Overall		DH		CHC-FRU	
	%	n	%	n	%	n
**Individual level**						
Age	*p=0.042*		*p=0.168*		*p=0.342*	
<24 years	23.9	1014	20.6	741	32.6	273
25-29 years	28.5	1145	24.6	805	37.6	340
30+ years	24.8	439	21.8	312	32.3	127
Missing	-	-	-	-	-	-
Total	26.1	2603	22.6	1860	35.0	743
Religion	*p=0.411*		*p=0.434*		*p=0.524*	
Hindu	26.4	2106	22.9	1519	35.4	587
Non-Hindu	24.6	492	20.9	339	32.7	153
Missing	-	-	-	-	-	-
Total	26.1	2603	22.6	1860	35.0	743
Caste	*p=0.809*		*p=0.461*		*p=0.375*	
SC/ST	26.9	692	23.9	486	34.0	206
OBC	26.0	1272	21.3	892	37.1	380
Other	25.3	597	23.6	454	30.8	143
Missing/DK	-	-	-	-	-	-
Total	26.1	2603	22.6	1860	35.0	743
Education	*p=0.374*		*p=0.437*		*p=0.100*	
<5 years	24.6	981	21.5	656	30.8	325
5-10 years	26.5	671	21.8	491	39.4	180
10+ years	27.3	945	24.1	711	37.2	234
DK/Missing	-	-	-	-	-	-
Total	26.1	2603	22.6	1860	35.0	743
Gravida	*p=0.302*		*p=0.327*		*p=0.849*	
1	24.0	757	21.1	563	32.5	194
2	27.4	781	24.2	575	36.4	206
3	27.8	623	24.1	431	35.9	192
4+	24.8	436	19.7	289	34.7	147
DK/Missing	-	-	-	-	-	-
Total	26.1	2603	22.6	1860	35.0	743
